# Supplementary material for: Unfavorable outcomes to second-line tuberculosis therapy among HIV-infected versus HIV-uninfected patients in sub-Saharan Africa: A systematic review and meta-analysis
Source: PLoS One. 2020 Aug 14;15(8):e0237534. doi: 10.1371/journal.pone.0237534 (PMC7428180; doi:10.1371/journal.pone.0237534)
Supplement: S1 Table — (DOCX) [file pone.0237534.s001.docx]

**S1 Table: Quality assessment for the included studies in meta-analysis.**

| **References** | **JBI’s Critical Appraisal Checklist** | | | | | | | | | | | **Score (Yes)** |
| --- | --- | --- | --- | --- | --- | --- | --- | --- | --- | --- | --- | --- |
|  | **Q1** | **Q2** | **Q3** | **Q4** | **Q5** | **Q6** | **Q7** | **Q8** | **Q9** | **Q10** | **Q11** |  |
| Adewumi et al, 2012 | Yes | NA | Yes | No | No | Yes | Yes | Yes | Yes | No | No | 6 |
| Alakaye et al, 2018 | NA | NA | Yes | No | No | Yes | Yes | Yes | Yes | No | Yes | 6 |
| Alene et al, 2017 | NA | NA | Yes | Yes | No | Yes | Yes | Yes | Yes | No | Yes | 7 |
| Brust et al, 2018 | NA | NA | Yes | Yes | No | Yes | Yes | Yes | Yes | No | Yes | 7 |
| Cox et al, 2014 | NA | NA | Yes | Yes | No | Yes | Yes | Yes | Yes | Yes | Yes | 8 |
| Farley et al, 2011 | Yes | UC | Yes | Yes | No | Yes | Yes | No | Yes | No | Yes | 7 |
| Hall et al, 2017 | NA | NA | Yes | Yes | No | Yes | Yes | Yes | Yes | No | Yes | 7 |
| Huerga et al, 2017 | NA | NA | Yes | Yes | No | Yes | No | Yes | Yes | No | Yes | 6 |
| Jikijela et al, 2018 | NA | NA | Yes | Yes | No | Yes | Yes | Yes | No | No | Yes | 6 |
| Ketema et al, 2019 | NA | NA | Yes | Yes | No | Yes | Yes | Yes | Yes | No | Yes | 7 |
| Leveri et al, 2019 | NA | NA | Yes | Yes | No | Yes | Yes | Yes | Yes | No | Yes | 7 |
| Loveday et al, 2015 | Yes | UC | Yes | Yes | No | Yes | Yes | Yes | Yes | No | Yes | 8 |
| Marais et al, 2014 | NA | NA | Yes | Yes | No | Yes | Yes | Yes | Yes | No | Yes | 7 |
| Mengistu et al, 2019 | NA | NA | Yes | Yes | Yes | Yes | Yes | Yes | Yes | No | Yes | 8 |
| Meresa et al, 2015 | NA | NA | Yes | Yes | Yes | No | Yes | Yes | Yes | No | Yes | 7 |
| Mohr et al, 2015 | NA | NA | Yes | Yes | No | Yes | Yes | Yes | Yes | No | Yes | 7 |
| Piubello et al, 2020 | NA | NA | Yes | Yes | No | Yes | Yes | Yes | Yes | Yes | Yes | 8 |
| Satti et al, 2012 | NA | NA | Yes | Yes | No | Yes | Yes | Yes | Yes | No | Yes | 7 |
| Shin et al, 2017 | NA | NA | Yes | Yes | No | Yes | Yes | Yes | Yes | Yes | Yes | 8 |

**Note**: NA, not applicable; UN, unclear; Q1-11, JBI’s Critical Appraisal Checklist for Cohort studies {Q1: Were the two groups similar and recruited from the same population? Q2: Were the exposures measured similarly to assign people to both exposed and unexposed groups? Q3: Was the exposure measured in a valid and reliable way? Q4: Were confounding factors identified? Q5: Were strategies to deal with confounding factors stated? Q6: Were the groups/participants free of the outcome at the start of the study (or at the moment of exposure)? Q7: Were the outcomes measured in a valid and reliable way? Q8: Was the follow up time reported and sufficient to be long enough for outcomes to occur? Q9: Was follow up complete, and if not, were the reasons to loss to follow up described and explored? Q10: Were strategies to address incomplete follow up utilized? Q11: Was appropriate statistical analysis used?}.
