# Supplementary figures and images for: Unfavorable outcomes to second-line tuberculosis therapy among HIV-infected versus HIV-uninfected patients in sub-Saharan Africa: A systematic review and meta-analysis
Source: PLoS One. 2020 Aug 14;15(8):e0237534. doi: 10.1371/journal.pone.0237534 (PMC7428180; doi:10.1371/journal.pone.0237534)

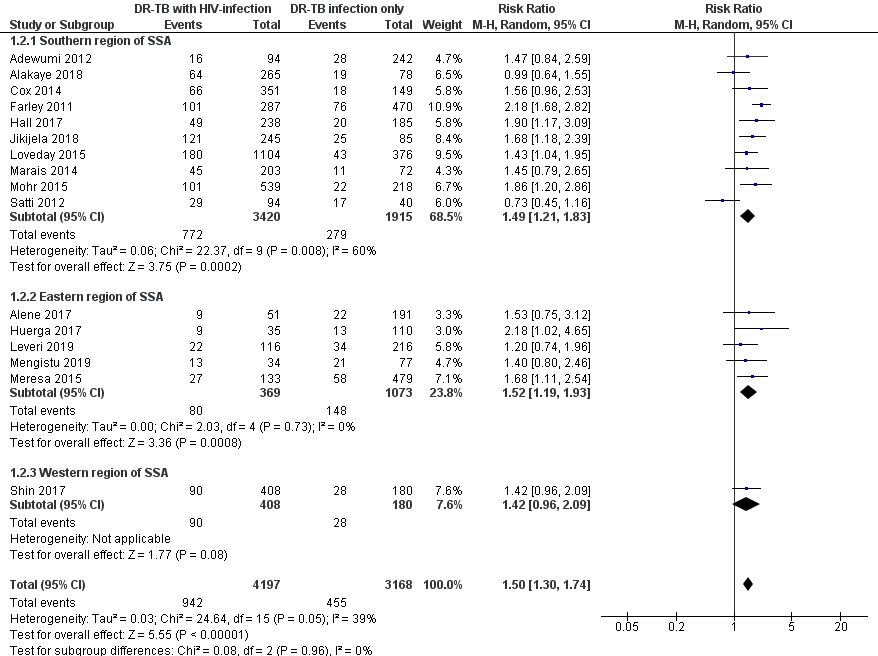

Supplement: S1 Fig — (TIF) [file pone.0237534.s003.tif]
