# Supplementary material for: Unfavorable outcomes to second-line tuberculosis therapy among HIV-infected versus HIV-uninfected patients in sub-Saharan Africa: A systematic review and meta-analysis
Source: PLoS One. 2020 Aug 14;15(8):e0237534. doi: 10.1371/journal.pone.0237534 (PMC7428180; doi:10.1371/journal.pone.0237534)
Supplement: S1 File — (DOCX) [file pone.0237534.s004.docx]

**Annex 2:** **Abbreviations (acronyms)**

CI Confidence Interval

DR-TB Drug-resistant Tuberculosis

HIV Human Immunodeficiency Virus

JBI Joanna Briggs Institute

MDR-TB Multidrug-resistant Tuberculosis

MeSH Medical Subject Heading

PRISMA Preferred Reporting Items for Systematic Review and Meta-Analysis

RR Risk Ratio

SSA sub-Saharan Africa

TB Tuberculosis

WHO World Health Organization
