# Supplementary material for: Unfavorable outcomes to second-line tuberculosis therapy among HIV-infected versus HIV-uninfected patients in sub-Saharan Africa: A systematic review and meta-analysis
Source: PLoS One. 2020 Aug 14;15(8):e0237534. doi: 10.1371/journal.pone.0237534 (PMC7428180; doi:10.1371/journal.pone.0237534)
Supplement: Attachment — Submitted filename: PONE-D-20-11009.docx [file pone.0237534.s005.docx]

Dear editors,

Please find my review of the manuscript (PONE-D-20-11009) entitled “Unfavorable outcomes to second-line tuberculosis therapy among HIV-infected versus HIV-uninfected patients in sub-Saharan Africa: a meta-analysis of individual patient data” by Dumessa Edessa et al.

In this systematic review article, the authors estimated the risks of unsuccessful outcomes (death, treatment failure, and loss from treatment) to second-line tuberculosis therapy in HIV-infected versus HIV-uninfected patients with drug-resistant tuberculosis pooled from 19 eligible studies. Meta-analysis was performed on 4481 HIV-infected and 3820 HIV uninfected African DR-TB patients, 1766 and 1164 patients having unfavorable outcomes, respectively. They found a higher risk ratio (RR) of overall unfavorable outcomes and death in HIV-infected patients. In addition, subgroup analysis showed a higher RR of unfavorable outcomes in mixed-age and in the eastern region of sub-Saharan Africa.

The systematic review and meta-analysis were done properly and there are no major concerns. Below are some minor concerns:

1) Did the DR-TB patients include MDR-TB patients?
2) Were all the HIV-infected TB patients on ART? How ART status might affect RR for death and overall unfavorable outcomes? Please list ART status in Table 1 and discuss how this could affect the RR for death and overall unfavorable outcomes.
3) When reporting overall significance, please use the exact p value instead of P>0.05 or <0.05.
4) Was the RR for death higher in the eastern region of SSA?
5) It is not clear what the significance of a higher RR in mixed age HIV-infected and uninfected patients. As RR in the adult and adolescent subgroup was not significantly different between HIV-infected and uninfected patients, is the difference in the RR for the overall unsuccessful outcomes mainly from pediatric patients?
6) In sensitivity and subgroup analyses, please provide description of outliners
7) The overall z score and p value need to be added to the Sensitivity and subgroup analyses section.
8) The figures are of very low quality and are illegible.
