# Supplementary material for: Unfavorable outcomes to second-line tuberculosis therapy among HIV-infected versus HIV-uninfected patients in sub-Saharan Africa: A systematic review and meta-analysis
Source: PLoS One. 2020 Aug 14;15(8):e0237534. doi: 10.1371/journal.pone.0237534 (PMC7428180; doi:10.1371/journal.pone.0237534)
Supplement: Attachment — Submitted filename: Response to reviewers comments.docx [file pone.0237534.s006.docx]

PONE-D-20-11009
Unfavorable outcomes to second-line tuberculosis therapy among HIV-infected versus HIV-uninfected patients in sub-Saharan Africa: a systematic review and meta-analysis

PLOS ONE

Journal requirements:

When submitting your revision, we need you to address these additional requirements.

 1. Please ensure that your manuscript meets PLOS ONE's style requirements, including those for file naming. The PLOS ONE style templates can be found at

https://journals.plos.org/plosone/s/file?id=wjVg/PLOSOne_formatting_sample_main_body.pdf and

https://journals.plos.org/plosone/s/file?id=ba62/PLOSOne_formatting_sample_title_authors_affiliations.pdf

Response: Thank you. Our manuscript meets PLOS ONE’s style requirements. Please check it.

2. Please include a discussion of how/whether you searched for unpublished studies (e.g. grey literature) in order to reduce the impact of publication bias on your results.

Response: Thank you so much for your comment. We made searches for unpublished studies through Google scholar which also links contents from ResearchGate. For example, four of the studies finally included in our meta-analysis were unpublished literature retrieved from institutional repositories of universities through Google scholar. So, a similar explanation is addressed in methods and publication bias section of the results in the revised manuscript.

3. We suggest you thoroughly copyedit your manuscript for language usage, spelling, and grammar. If you do not know anyone who can help you do this, you may wish to consider employing a professional scientific editing service.  
Whilst you may use any professional scientific editing service of your choice, PLOS has partnered with both American Journal Experts (AJE) and Editage to provide discounted services to PLOS authors. Both organizations have experience helping authors meet PLOS guidelines and can provide language editing, translation, manuscript formatting, and figure formatting to ensure your manuscript meets our submission guidelines. To take advantage of our partnership with AJE, visit the AJE website (http://learn.aje.com/plos/) for a 15% discount off AJE services. To take advantage of our partnership with Editage, visit the Editage website (www.editage.com) and enter referral code PLOSEDIT for a 15% discount off Editage services.  If the PLOS editorial team finds any language issues in text that either AJE or Editage has edited, the service provider will re-edit the text for free.

Response: Thank you for your concern. We do not have significant English language problems and our revised manuscript is thoroughly edited for language usage, spelling, and grammar. Again, we are happy to hear from you (i.e., reviewers/editor) regarding any more correction requirements that may specifically be indicated for English language errors in the revised manuscript.

Reviewers' comments:

Reviewer's Responses to Questions

**Comments to the Author**

1. Is the manuscript technically sound, and do the data support the conclusions?

The manuscript must describe a technically sound piece of scientific research with data that supports the conclusions. Experiments must have been conducted rigorously, with appropriate controls, replication, and sample sizes. The conclusions must be drawn appropriately based on the data presented.

Reviewer #1: Yes

Reviewer #2: Yes

2. Has the statistical analysis been performed appropriately and rigorously?

Reviewer #1: Yes

Reviewer #2: Yes

3. Have the authors made all data underlying the findings in their manuscript fully available?

The [PLOS Data policy](http://www.plosone.org/static/policies.action#sharing) requires authors to make all data underlying the findings described in their manuscript fully available without restriction, with rare exception (please refer to the Data Availability Statement in the manuscript PDF file). The data should be provided as part of the manuscript or its supporting information, or deposited to a public repository. For example, in addition to summary statistics, the data points behind means, medians and variance measures should be available. If there are restrictions on publicly sharing data—e.g. participant privacy or use of data from a third party—those must be specified.

Reviewer #1: Yes

Reviewer #2: Yes

 4. Is the manuscript presented in an intelligible fashion and written in standard English?

PLOS ONE does not copyedit accepted manuscripts, so the language in submitted articles must be clear, correct, and unambiguous. Any typographical or grammatical errors should be corrected at revision, so please note any specific errors here.

Reviewer #1: Yes

Reviewer #2: Yes

5. Review Comments to the Author

Please use the space provided to explain your answers to the questions above. You may also include additional comments for the author, including concerns about dual publication, research ethics, or publication ethics. (Please upload your review as an attachment if it exceeds 20,000 characters)

Reviewer #1: Drug-resistant tuberculosis (DR-TB), including multidrug-resistant TB (MDR-TB) and extensively drug-resistant TB (XDR-TB), is considered a potential obstacle for elimination of TB globally. HIV coinfection with DR-TB further complicates the scenario. HIV-1 comanagement with ART could worsen outcomes of second-line anti-tuberculosis drugs.
In this study, authors measured the risk ratios for the unfavorable outcomes in HIV-infected TB patients compared with HIV-uninfected TB patient by analyzing 19 studies. They found both a higher risk of an unfavorable TB treatment outcome and higher risk of death in HIV-infected patients. Their results indicate that special strategies that reduce the risk of death should be discovered and implemented to treat HIV-infected patients with second-line anti-TB therapy.
I recommend to accept this manuscript for publication.

Reviewer #2: In this systematic review article, the authors estimated the risks of unsuccessful outcomes (death, treatment failure, and loss from treatment) to second-line tuberculosis therapy in HIV-infected versus HIV-uninfected patients with drug-resistant tuberculosis pooled from 19 eligible studies. Meta-analysis was performed on 4481 HIV-infected and 3820 HIV uninfected African DR-TB patients, 1766 and 1164 patients having unfavorable outcomes, respectively. They found a higher risk ratio (RR) of overall unfavorable outcomes and death in HIV-infected patients. In addition, subgroup analysis showed a higher RR of unfavorable outcomes in mixed-age and in the eastern region of sub-Saharan Africa.
The systematic review and meta-analysis were done properly and there are no major concerns. Below are some minor concerns:
1) Did the DR-TB patients include MDR-TB patients?

Response to reviewer: Thank you very much for your comments. The DR-TB patients we considered also includes MDR-TB patients. Please see our data search strategy and inclusion/exclusion criteria subsections under methods section of the manuscript for your confirmation.

2) Were all the HIV-infected TB patients on ART? How ART status might affect RR for death and overall unfavorable outcomes? Please list ART status in Table 1 and discuss how this could affect the RR for death and overall unfavorable outcomes.

Response to reviewer: Thank you for your concerns. All the included studies narrate that HIV-infected patients were either initiated with ART or on pre-ART. Unfortunately, none of the studies report treatment outcomes (i.e., death, treatment failure, or loss from treatment) by the ART status. Again, this approach addresses the HIV-infected DR-TB patients only and it misses our aim of comparing unfavorable treatment outcomes between HIV-infected and HIV-uninfected DR-TB patients. So, since we did not plan and search for literature in consideration of this question, we don’t have data required to estimate the RR for death and overall unfavorable outcomes among HIV-infected patients treated with second-line anti-TB therapy by their ART status. Moreover, it is not sufficiently feasible to address this concern under our current searches and objective in this systematic review and meta-analysis. We found that addressing this concern necessitates another methodology for potential literature searches, screening processes and eligibility assessments for us to provide a rational scientific analysis and explanation. That means, we recommend another study (meta-analysis) that will be planned with a specific search strategy as a potential scientific evidence to justify the present concern as already raised.

3) When reporting overall significance, please use the exact p value instead of P>0.05 or <0.05.

Response to reviewer: Thank you so much, we have incorporated the exact p-value for all p-values in our analysis result. Please check the results section of the revised manuscript.

4) Was the RR for death higher in the eastern region of SSA?

Response to reviewer: Thank you for your comment. The RR for death during second-line TB therapy among HIV-infected versus HIV-uninfected patients was 1.52 (95% CI: 1.19-1.93; I^2^ = 0%; P=0.73; z=3.36; P=0.0008) which is still slightly higher than the estimates for the other regions. There were increased RR estimates for all regions of the SSA. And none of them revealed differences from the overall RR estimate for death (RR=1.50; 95% CI: 1.30 – 1.74; I^2^ = 39%; P=0.05) and we included this finding in supplementary information. So, detailed information can be obtained from S1 Figure in supplementary information section.

5) It is not clear what the significance of a higher RR in mixed age HIV-infected and uninfected patients. As RR in the adult and adolescent subgroup was not significantly different between HIV-infected and uninfected patients, is the difference in the RR for the overall unsuccessful outcomes mainly from pediatric patients?

Response to reviewer: Thank you for your concerns. Indeed, we reported what we found by subgroup analyses in an attempt to decrease the degree of heterogeneity among the included studies. As already raised, pediatric and elderly individuals were part of the mixed-age patients in whom immune statuses might be incompetent compared with adult/adolescent counterparts. This could be related with immature immune function in children and reduced immune function with an aged physiology in elderly. More specifically, treatment successes for both HIV and TB infections require optimal function of the immune cells. The immune functions of such individuals are further weakened in the presence of the HIV and TB co-infections compared with the TB infection alone. So, the increased RR estimate for the overall unfavorable outcome in mixed-age patients treated with second-line TB therapy among HIV-infected versus HIV-uninfected patients could indicate these differences in age-related physiologic immaturity and/or changes and additive effects of both infections. These scientific explanations are included in discussion section of the revised manuscript.

6) In sensitivity and subgroup analyses, please provide description of outliers.

Response to reviewer: Thank you for your comment. Description of outliers as those studies with far lower or higher values of the RR estimate for the unfavorable outcomes than the pooled RR estimate for the outcome of interest is included under sensitivity and subgroup analyses of the results section in the revised manuscript. Details of the studies we considered as outliers were also cited in the manuscript. Please check this revision in results section of the manuscript.

7) The overall z score and p value need to be added to the Sensitivity and subgroup analyses section.

Response to reviewer: Thank you. This issue is addressed in result section of the revised manuscript.

8) The figures are of very low quality and are illegible.

Response to reviewer: Thank you for your concern. All figures are RevMan generated and we can do nothing manually. However, we checked resolution of each figure before submission by the use of PACE and all figures are PACE-corrected. Indeed, each figure is visible if opened individually. However, merged figures (i.e., Fig 6A-B and Fig 7A-D) might have poor visibility due to the journal requirement to merge section figures as a single figure that should be displayed in a separate page. If the journal allows us to make Figs 6A-B and 7A-D as separate figures, their resolution and visibility can be further improved.

 6. PLOS authors have the option to publish the peer review history of their article ([what does this mean?](https://journals.plos.org/plosone/s/editorial-and-peer-review-process#loc-peer-review-history)). If published, this will include your full peer review and any attached files.

If you choose “no”, your identity will remain anonymous but your review may still be made public.

**Do you want your identity to be public for this peer review?** For information about this choice, including consent withdrawal, please see our [Privacy Policy](https://www.plos.org/privacy-policy).

Reviewer #1: No

Reviewer #2: No

[NOTE: If reviewer comments were submitted as an attachment file, they will be attached to this email and accessible via the submission site. Please log into your account, locate the manuscript record, and check for the action link "View Attachments". If this link does not appear, there are no attachment files.]

While revising your submission, please upload your figure files to the Preflight Analysis and Conversion Engine (PACE) digital diagnostic tool, <https://pacev2.apexcovantage.com/>. PACE helps ensure that figures meet PLOS requirements. To use PACE, you must first register as a user. Registration is free. Then, login and navigate to the UPLOAD tab, where you will find detailed instructions on how to use the tool. If you encounter any issues or have any questions when using PACE, please email PLOS at [figures@plos.org](mailto:figures@plos.org). Please note that Supporting Information files do not need this step.
